# Use of oblique sagittal and coronal weighted images for diagnosis and grading of ACL graft injury

**DOI:** 10.1186/s43055-022-00790-4

**Published:** 2022-05-24

**Authors:** Mennatallah ElSayed, Amr Samir Rashwan, Heba Kamal

**Affiliations:** 1 Department of Diagnostic and Interventional Radiology, Kasr Al-Aini Faculty of Medicine, 8 Ahmed eElshediak street, Ard Elgolf, Heliopolis, Cairo Egypt; 2 Department of Orthopedics, Kasr Al-Ainy Faculty of Medicine, Heliopolis, Egypt

**Keywords:** ACL graft, Magnetic resonance imaging, Arthroscopy

## Abstract

**Background:**

This study was done to evaluate the value of adding the oblique sagittal and oblique coronal MRI to the standard MRI knee protocol for evaluation of suspected ACL graft injuries.

**Results:**

This was a cross-sectional analytic study where we reviewed 36 MRI knee examinations of 36 patients (30 males, 6 females, age range: 17–60 years, mean age: 26 years) who were subjected to ACL reconstruction and follow-up arthroscopy. Two experienced radiologists, blinded to the results of each other, evaluated the status and the severity of the ACL graft injury using the routine knee MRI (protocol A) and using the routine MRI after adding the oblique sagittal and coronal imaging (protocol B). Weighted kappa statistics were used to evaluate the diagnostic accuracies of the knee MRI before and after the addition of the oblique sagittal and coronal weighted images (protocol A and protocol B, respectively) and to assess the interobserver agreement. The weighted kappa values according to the routine knee MRI were 0.357 (reader 1) and 0.399 (reader 2). The inclusion of additional oblique coronal imaging increased the weighted kappa values to 0.505 (reader 1) and 0.528 (reader 2). The interobserver agreement weighted kappa value also increased from 0.606 to 0.759 by adding the oblique sagittal and coronal imaging to the routine knee MRI examination.

**Conclusion:**

The additional use of oblique sagittal and coronal MRI of the knee improves the diagnostic accuracy for diagnosing and grading ACL graft injury with the arthroscopy used as a gold standard.

## Background

- In the field of ACL injury and reconstruction, magnetic resonance imaging (MRI) represents a useful preoperative tool to confirm a disruption of the ACL. MRI is also valuable post-operatively to assess graft healing and maturation, to determine its position and to evaluate potential complications or re-injury [1–3].
- On the other hand, arthroscopy is another diagnostic method which allows direct visualization of all intraarticular structures [4]. However, arthroscopy is considered to be relatively expensive and invasive [5]; that is why the use of a new procedure with high diagnostic value in evaluating ACL graft injury is required.
- Most of the previous magnetic resonance studies have studied the status of anterior cruciate ligament grafts using orthogonal sagittal and coronal images. An ideal combination of slice orientation, thickness and pulse sequences may be needed, but results are still only suggestive of the injury [6].
- Few studies have studied the role of oblique coronal and sagittal images for evaluation and grading anterior cruciate ligament graft injuries [7, 8].
- For the native ACL, several studies have shown that an additional oblique view improves the diagnostic efficacy of ACL tears with respect to specificity and accuracy [9–13]
- For ACL grafting, Moon et al. [8] have shown that the additional oblique coronal view of the MRI of the knee improves both the diagnostic accuracy and confidence for grading ACL graft injury.
- Kiekara et al. [15] included both oblique sagittal and coronal views for evaluation of reconstructed ACL.
- The aim of our study was to determine the additive value of using oblique sagittal and coronal MRI for evaluation and grading of injury of ACL graft with the arthroscopy used as a gold standard.

## Methods

### Patients

Approval of the ethics committee was obtained for this cross-sectional analytic study. We reviewed 36 patients (30 males, 6 females, age range: 17–60 years, mean age: 26 years) with history of anterior cruciate ligament reconstruction. The frequency and percentage of patients according to sex and side of knee affection in the study population are shown in Table 1. The patients had persistent or recurrent symptoms or had re-injury of their knee. The patients were recruited from Orthopedic Department of Kasr El Ainy Hospital, Faculty of Medicine, Cairo University. All patients were subjected to history taking, clinical provisional and MRI examination of the affected knee joint. Arthroscopic examination of the affected knee joint was also done for all patients and considered as our gold standard. The MRI examinations of 35 patients were performed at a mean time of 8 months after initial ACL reconstruction surgery with a single patient's examination done 3 months after ACL reconstruction surgery. Patients who were excluded from the study include those with absolute contraindications to MR examination as cardiac pacemaker, aneurysmal clipping and claustrophobia, patients with knee bone tumors, patients with osteomyelitis, chronic muscle disorders, known active articular infection or metabolic bone diseases.

**Table 1:** Frequency and percentage according to gender and side of affected knee joint

	Count	%
*Gender*		
Female	6	16.7
Male	30	83.3
*Side*		
Left knee	15	41.7
Right knee	21	58.3

#### MRI protocol

**Fig. 1:**
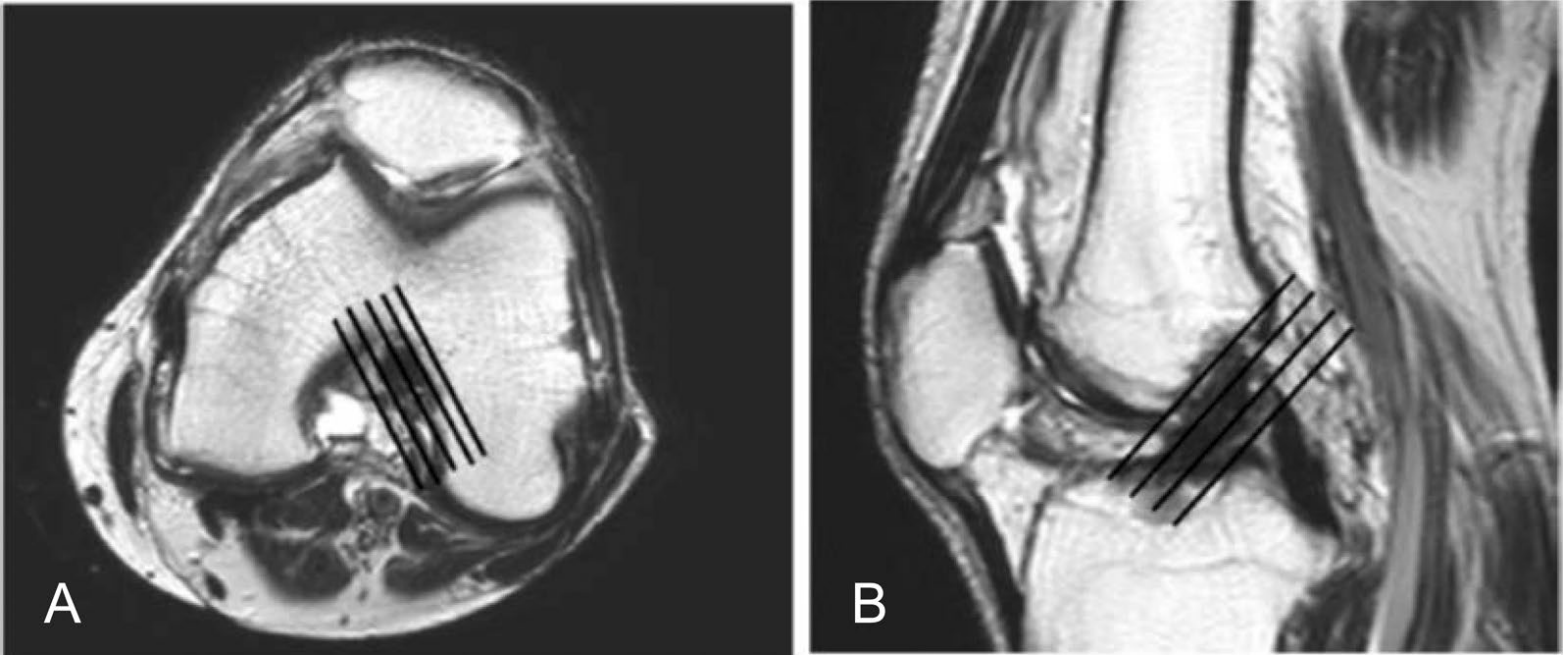
Oblique sagittal and coronal imaging of ACL was planned using both axial (**A**) and sagittal (**B**) images, respectively (black lines)

MR examinations were performed using an “ACHIEVA 1.5-Telsa equipment (from PHILIPS Medical Systems, Best, The Netherlands)” utilizing a phased array knee coil, at the Radiology Department of Kasr El Ainy Hospital, Cairo University. MRI examinations were done with the candidate lying in supine position with the joint space in the middle of the coil, while the knee joint was maintained in extension with slight flexion. Preliminary scout localizers in axial, coronal and sagittal planes were done. The standard knee protocol and the additional oblique sagittal and coronal sequences with their parameters are demonstrated in Table 2.The coverage should include all the anterior, posterior, medial and lateral supporting structures of the knee. Cranially, the distal aspect of the quadriceps tendon should be involved. The distal insertions of the patellar tendon should be included inferiorly. The oblique sagittal T2 planes are the planes on the axial localizer images that were 15 degrees from perpendicular to the bicondylar line (Fig. 1a), while the oblique coronal MRI protocol T2-FSE Images were obtained in the plane parallel to the ACL and the roof of the intercondylar notch (Blumensaat’s line) in the mid-sagittal localizer (Fig. 1b).

**Table 2:** Protocol of the MRI

	TR	TE	FOV	SL	Gap	Matrix
*Standard*						
Sagittal PD(TSE)	5000	30	180	4.5	0.7	576 × 512
Coronal STIR	5054.4	30	160	3.5	0.7	512 × 256
Axial T2(TSE)	3632	100	170	5	o.7	256 × 192
Sagittal T2	3619.4	100	180	4.5	o.7	512 × 256
Sagittal STIR	3931.6	100	180	4	0.7	256 × 192
*Additional sequences*						
Additional sagittal oblique T2	3500	95	150	3	1	256 × 150
Additional coronal oblique T2	3500	95	150	3	1	256 × 150

#### MRI imaging analysis

The produced MR images were transported to a workstation using the Digital Imaging and Communications in Medicine (DICOM) format. The images were evaluated by two musculoskeletal radiologists each with more than 10 years of experience, blinded to the results of each other and blinded to the arthroscopic results. Each reader independently assessed each ACL graft using the standard magnetic resonance images (protocol A) and then using the standard knee MRI in addition to the oblique sagittal and coronal images (protocol B).

Firstly, the standard imaging planes of the knee were evaluated (protocol A) with each ACL graft classified as intact, partially torn or completely torn. Thereafter, these standard planes were evaluated together with oblique sagittal imaging and oblique coronal imaging of the ACL (protocol B) and each ACL graft was again classified as intact, partially torn or completely torn.

**Fig. 2:**
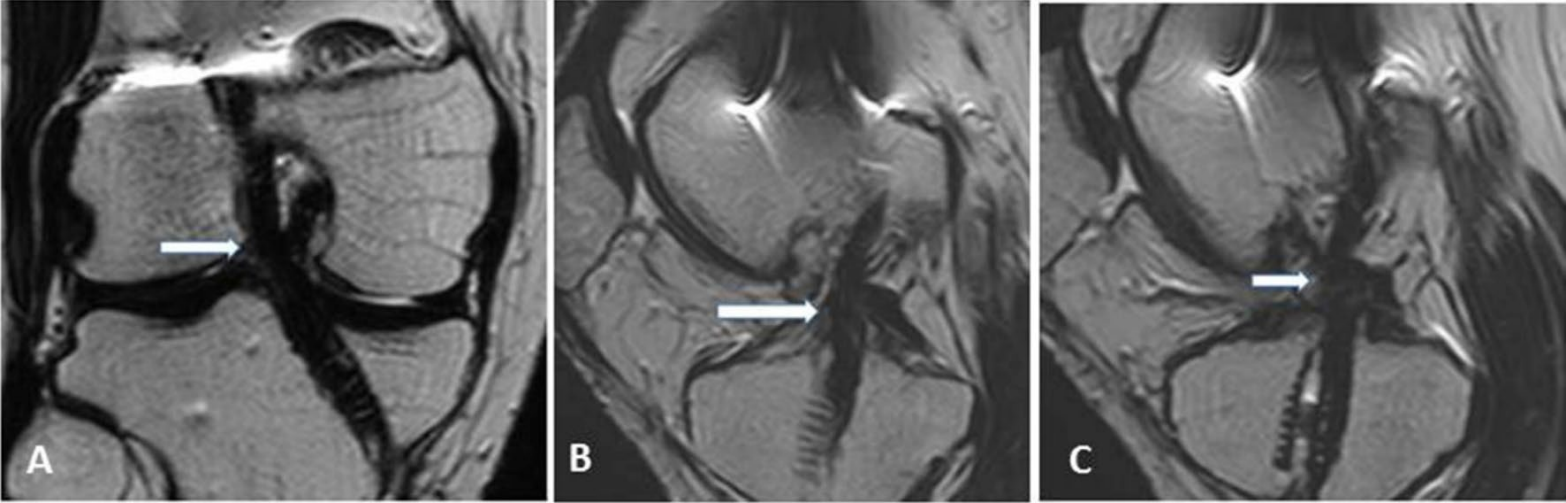
**a**, Coronal T2 WIs; **b** and **c**, sagittal T2 WIs showing an intact graft having a low signal intensity, well-preserved continuation and a taut orientation (thick white arrows)

**Fig. 3:**
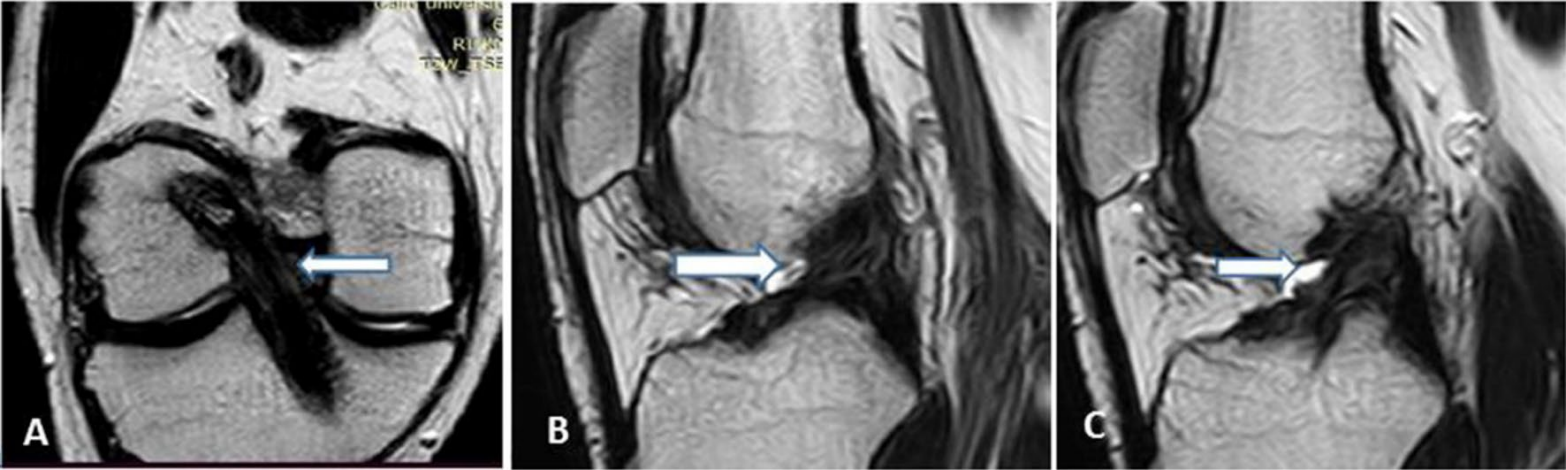
**a** Oblique coronal T2,** b** and** c** oblique sagittal T2 WIs showing interstitial high signal elevation, thinning at the middle portion of the graft with a slight lax orientation suggesting a grade 1 injury (white arrows)

**Fig. 4:**
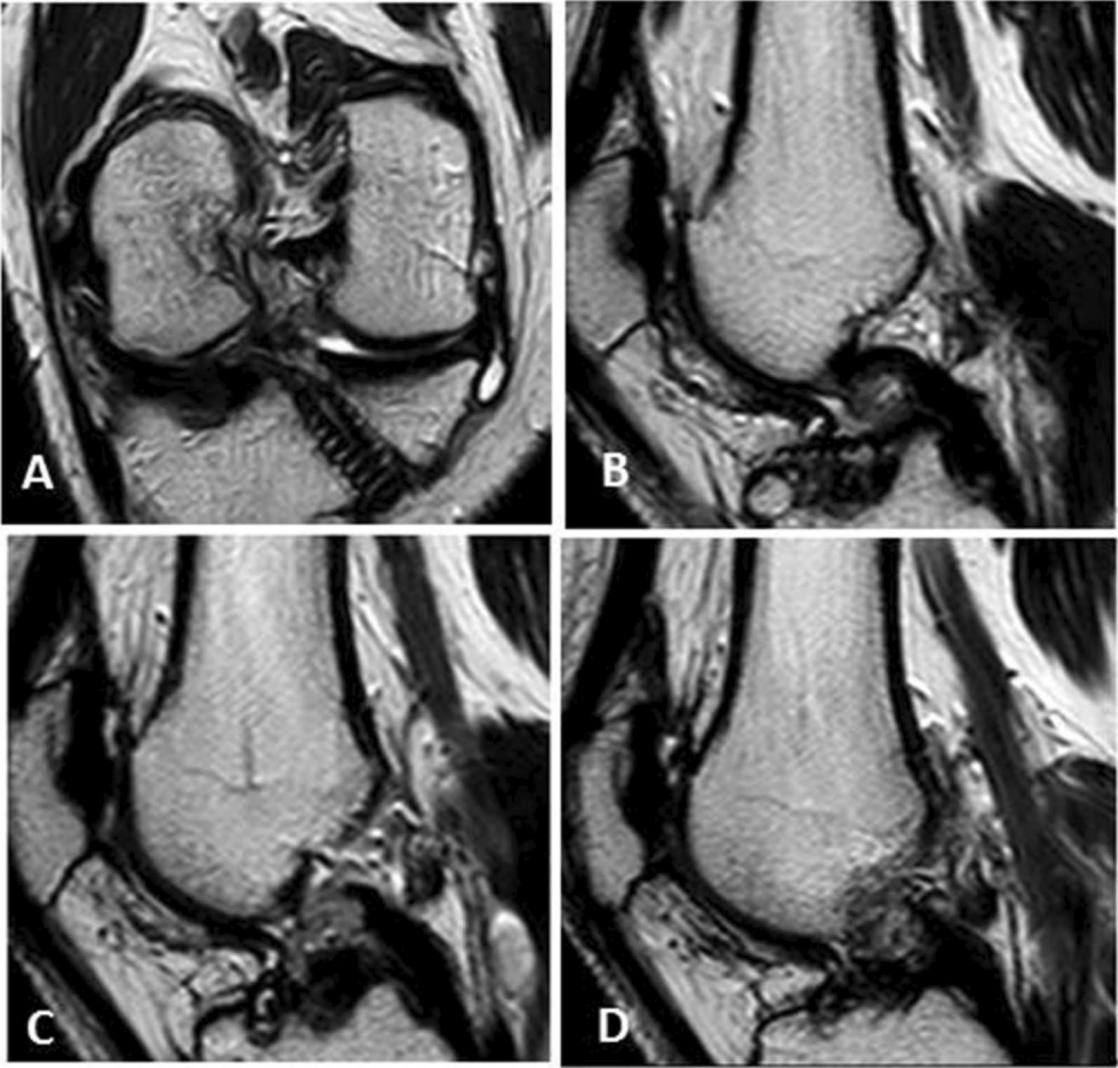
**a**, Oblique coronal and** b**,** c**,** d**, oblique sagittal T2WIs also showing indistinct ACL contour, suggesting grade 2 injury of anterior cruciate ligament graft

A three-point classification system was used to evaluate the grade of ACL graft injury: grades 0, 1 and 2. Grade 0 refers to an intact graft, grade 1 to a partial thickness tear and grade 2 to a complete tear of the ACL graft. In our study, we considered an intact graft as a low signal intensity graft with or without longitudinally increased signal intensity streaks, well-preserved continuation and stretched (Fig. 2). Some grafts with rarely diffuse or with focal increased signal intensity or a slight lax orientation were considered as intact grafts, while hyperintensities almost equal to fluid or graft thinning in the ACL grafts on T2-weighted images were regarded impressive of a partial or full thickness tear. To discriminate grade 1 from grade 2 injuries, a near full-thickness defect, a lack of continuity or an indistinct ligament contour was regarded suggestive of grade 2 injury (Figs. 3 and 4).

Arthroscopic examinations were done to all 36 patients. Arthroscopic reports were reviewed for assessment of the ACL graft. MRI results (standard knee protocol VS standard in addition to oblique sagittal and oblique coronal knee protocol) were compared with arthroscopic results with the arthroscopy used as a reference gold standard.

### Arthroscopic interpretation

Arthroscopic examinations were done by experienced orthopedic surgeons, with experience in knee joint arthroscopies of more than 10-year duration. At arthroscopy, each cruciate ligament graft was classified as being normal or having partial or complete tear.

#### Statistical analysis

Data were entered and statistically analyzed on the Statistical Package of Social Science Software program, version 25 (IBM SPSS Statistics for Windows, Version 25.0. Armonk, NY: IBM Corp.). Weighted kappa statistics were used to assess the diagnostic agreement between the MRI diagnoses and the arthroscopic results. The strength of interobserver agreement was interpreted according to the guidelines described by Landis and Koch [8], that is, 0: poor, 0.01–0.20: slight, 0.21–0.40: fair, 0.41–0.60: moderate, 0.61–0.80: substantial and 0.81–1.00: almost perfect. The sensitivity, specificity and accuracy for detecting partial and complete graft tear were calculated. P values less than or equal to 0.05 were considered statistically significant.

### The standard of reference

Knee arthroscopy was considered the gold-standard technique.

## Results

The MRI status and grades of the ACL graft injury concerning each reader and concerning each imaging protocol and diagnostic agreements between the MR grade and the arthroscopic grade are shown in Tables 3, 4, 5 and 6.The diagnostic agreements between the MR grade of injury and the arthroscopic grade of injury for imaging with protocol A were regarded as “**fair**” with weighted kappa values of 0.399 and 0.357 for reader 1 and reader 2, respectively. On the other hand, the diagnostic agreements between the MR grade of injury and the arthroscopic grade of injury for imaging with protocol B were regarded as “**moderate**” with weighted kappa values of 0.505 and 0.528 for reader 1 and reader 2, respectively.

**Table 3:** MRI grades of ACL graft injury in imaging protocol A versus arthroscopy according to reader 1. Diagnostic agreement between MR grade and arthroscopic grade is also included

Protocol A	Arthroscopy					
	Complete tear	Partial tear	No tear	Total	Kappa	95%CI
*Reader 1*						
Complete tear	7	3	0	10	0.399	0.163–0.634
Partial tear	2	3	6	11		
No tear	1	2	12	15		
Total	10	8	18	36		

*CI* Confidence interval

**Table 4:** MRI grades of ACL graft injury in imaging protocol A versus arthroscopy according to reader 2. Diagnostic agreement between MR grade and arthroscopic grade is also included

Protocol A	Arthroscopy					
	Complete tear	Partial tear	No tear	Total	kappa	95%CI
*Reader 2*						
Complete tear	6	1	0	7	0.357	0.108–0.606
Partial tear	2	2	4	8		
No tear	2	5	14	21		
Total	10	8	18	36		

*CI* Confidence interval

**Table 5:** MRI grades of ACL graft injury in imaging protocol B versus arthroscopy according to reader 1. Diagnostic agreement between MR grade and arthroscopic grade is also included

Protocol B	Arthroscopy					
	Complete tear	Partial tear	No tear	Total	Kappa	95%CI
*Reader 1*						
Complete tear	7	2	0	9	0.505	0.274–0.736
Partial tear	2	3	3	8		
No tear	1	3	15	19		
Total	10	8	18	36		

*CI* Confidence interval

**Table 6:** MRI grades of ACL graft injury in imaging protocol B versus arthroscopy according to reader 2. Diagnostic agreement between MR grade and arthroscopic grade is also included

Protocol B	Arthroscopy					
	Complete tear	Partial tear	No tear	Total	Kappa	95%CI
*Reader 2*						
Complete tear	7	1	0	8	0.528	0.301–0.754
Partial tear	2	2	1	5		
No tear	1	5	17	23		
Total	10	8	18	36		

*CI* Confidence interval

**Fig. 5:**
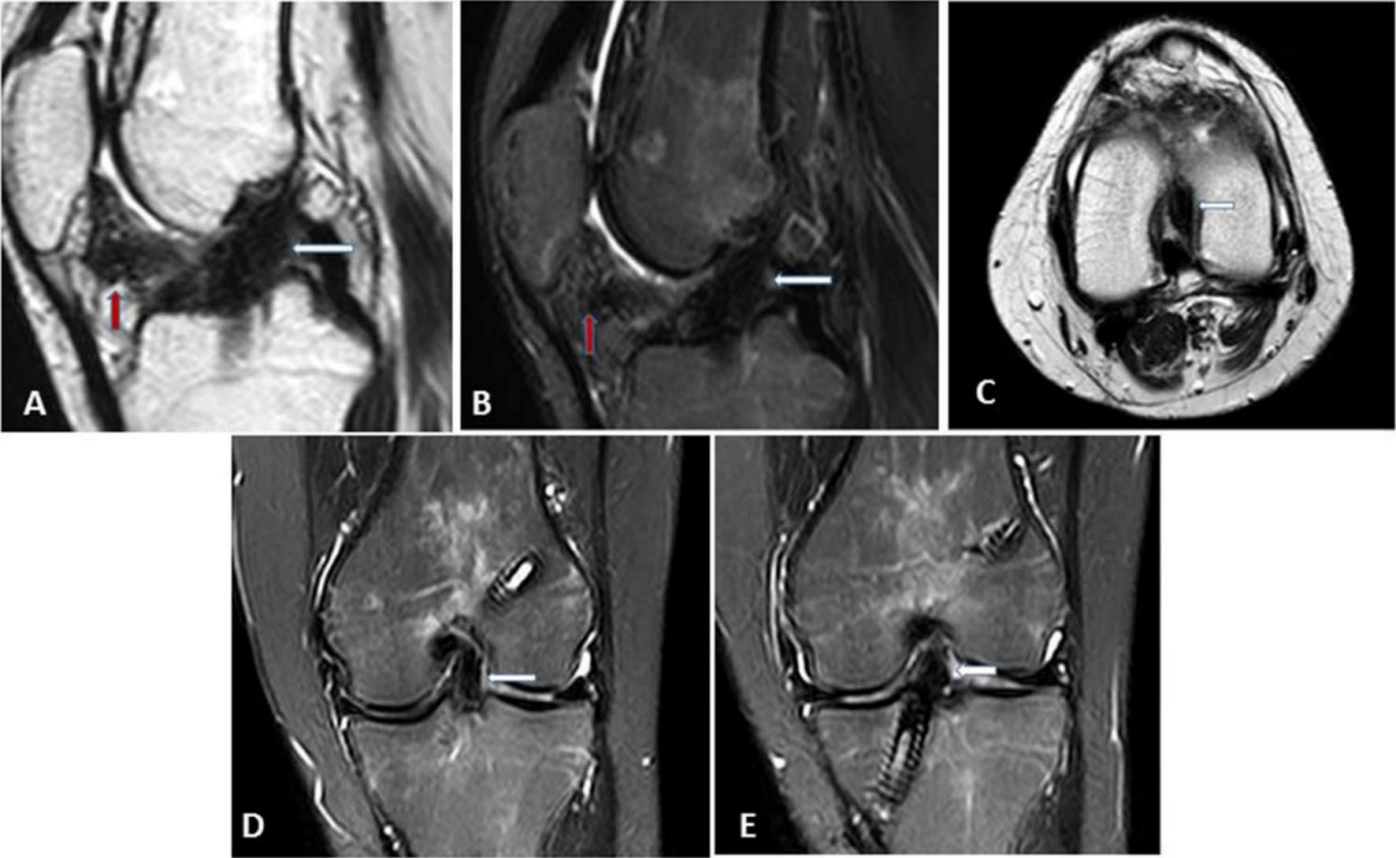
Female patient, 22 years old with left ACL reconstruction. She came now complaining of pain and limitation of movement. **A**, sagittal T2; **B**, sagittal T2 fat sat; **C**, axial T2; **D** and **E** coronal T2 fat sat WIs (protocol A) showing preserved diffuse low signal of the graft with proper graft inclination suggesting grade 0 injury (white thick arrows). Associated localized arthrofibrosis of Hoffa’s fat pad is also seen (thick red arrow)

**Fig. 6:**
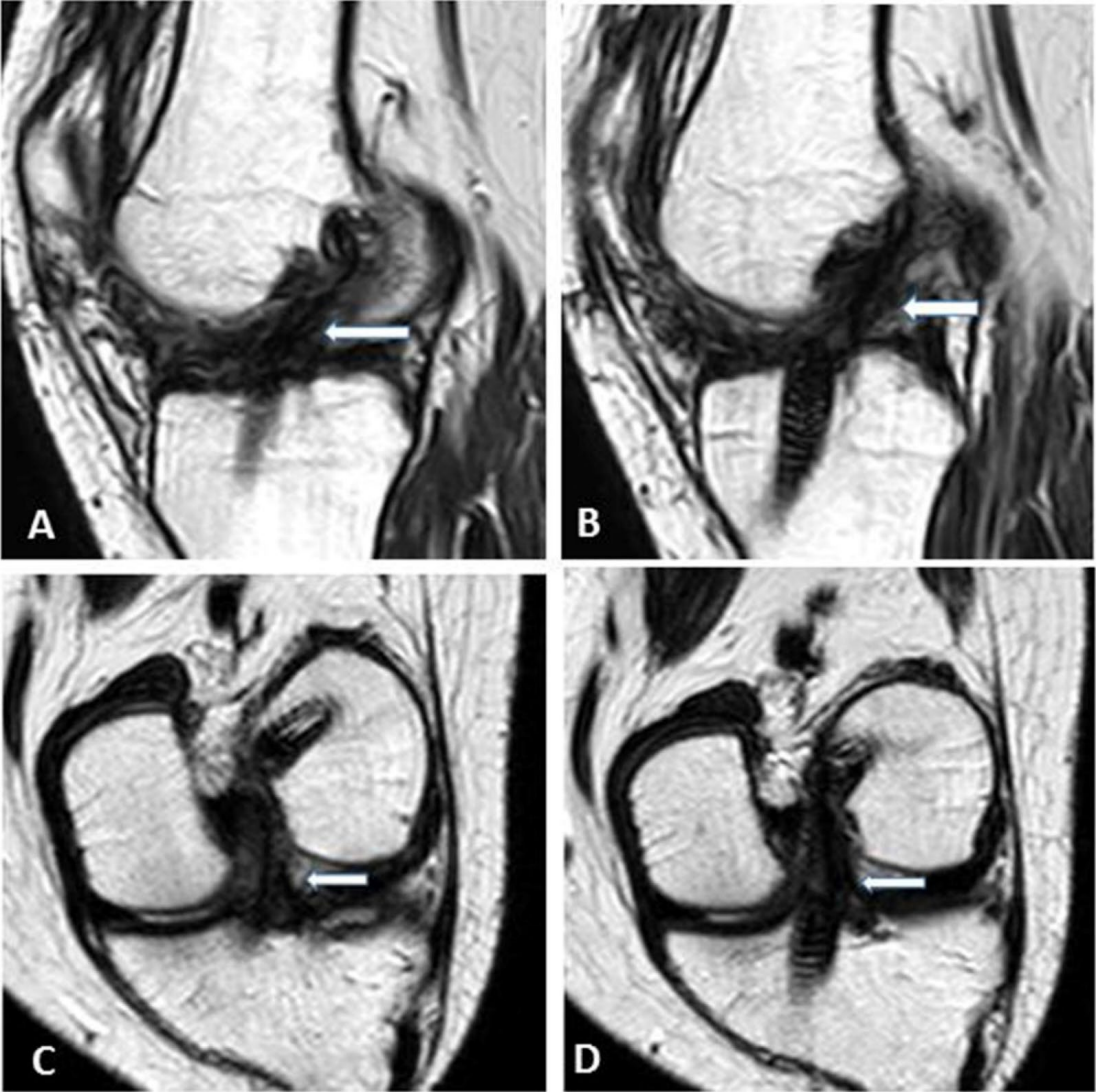
**a**, **b** sagittal oblique T2 WIs; **c** and **d** coronal oblique T2 WIs (protocol B) of the same patient mentioned in Fig. 5, showing interstitial high signal elevation and thinning at the middle and tibial portion of the graft suggesting a grade 1 injury rather than grade 0 (thick white arrows). Arthroscopic examination 8 months later confirmed grade 1 injury

Regarding protocol A, Reader 1 and arthroscopy had matching results in 22 cases: 7 cases with complete tear, 3 cases with partial tear and 12 cases with no tear. Reader 1 and arthroscopy disagreed in 14 cases. Reader 2 and arthroscopy agreed also in 22 patients:6 cases with complete tear, 2 cases with partial tear and no tear in 14 cases. Reader 2 and arthroscopy disagreed in 14 cases (Figs. 5, 6).

**Fig. 7:**
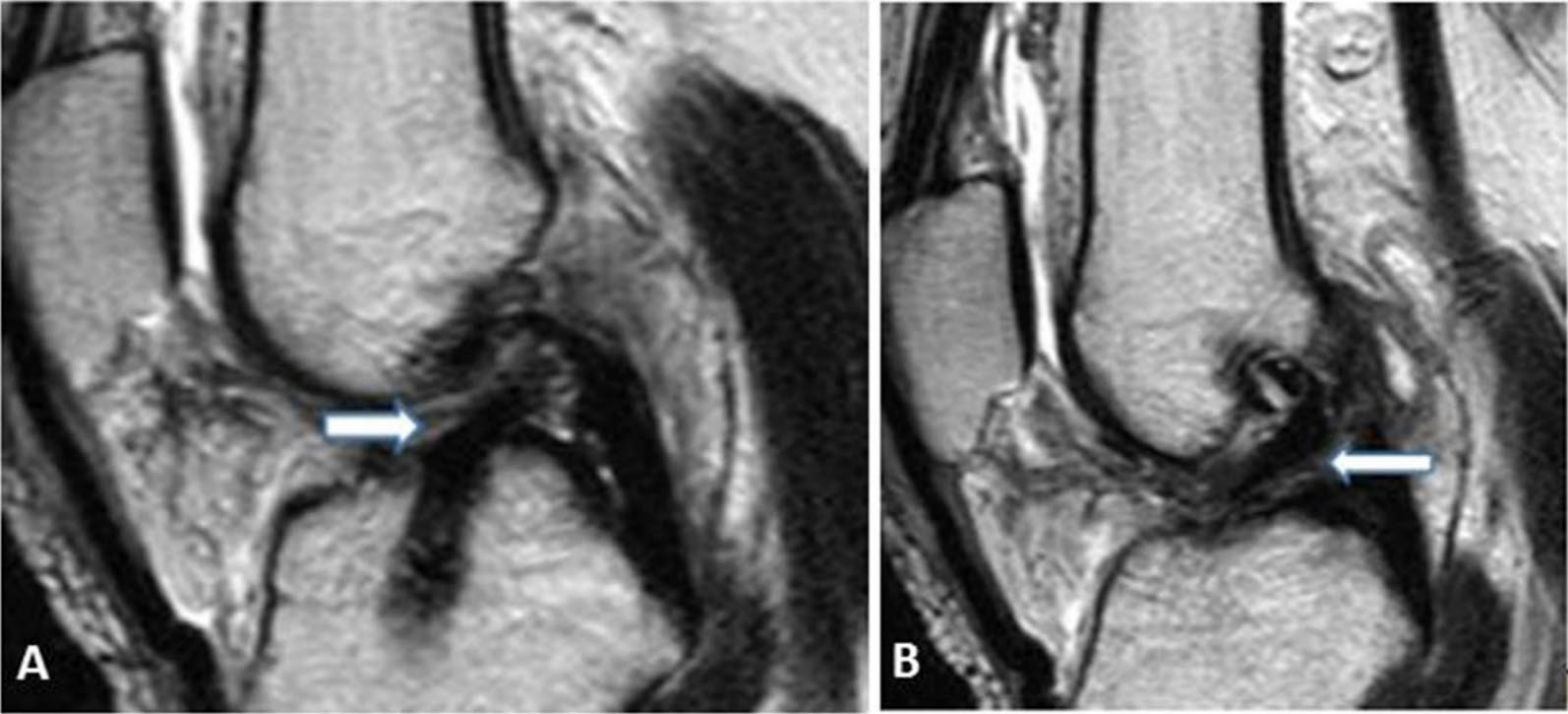
36-year-old male patient with history of right ACL reconstruction. He came now with right knee pain increasing with movement and knee instability.** A** and** B** sagittal T2WI (protocol A) showing lax orientation of the ACL graft fibers with fuzzy outline, suggesting grade 1 graft injury (thick white arrows)

**Fig. 8:**
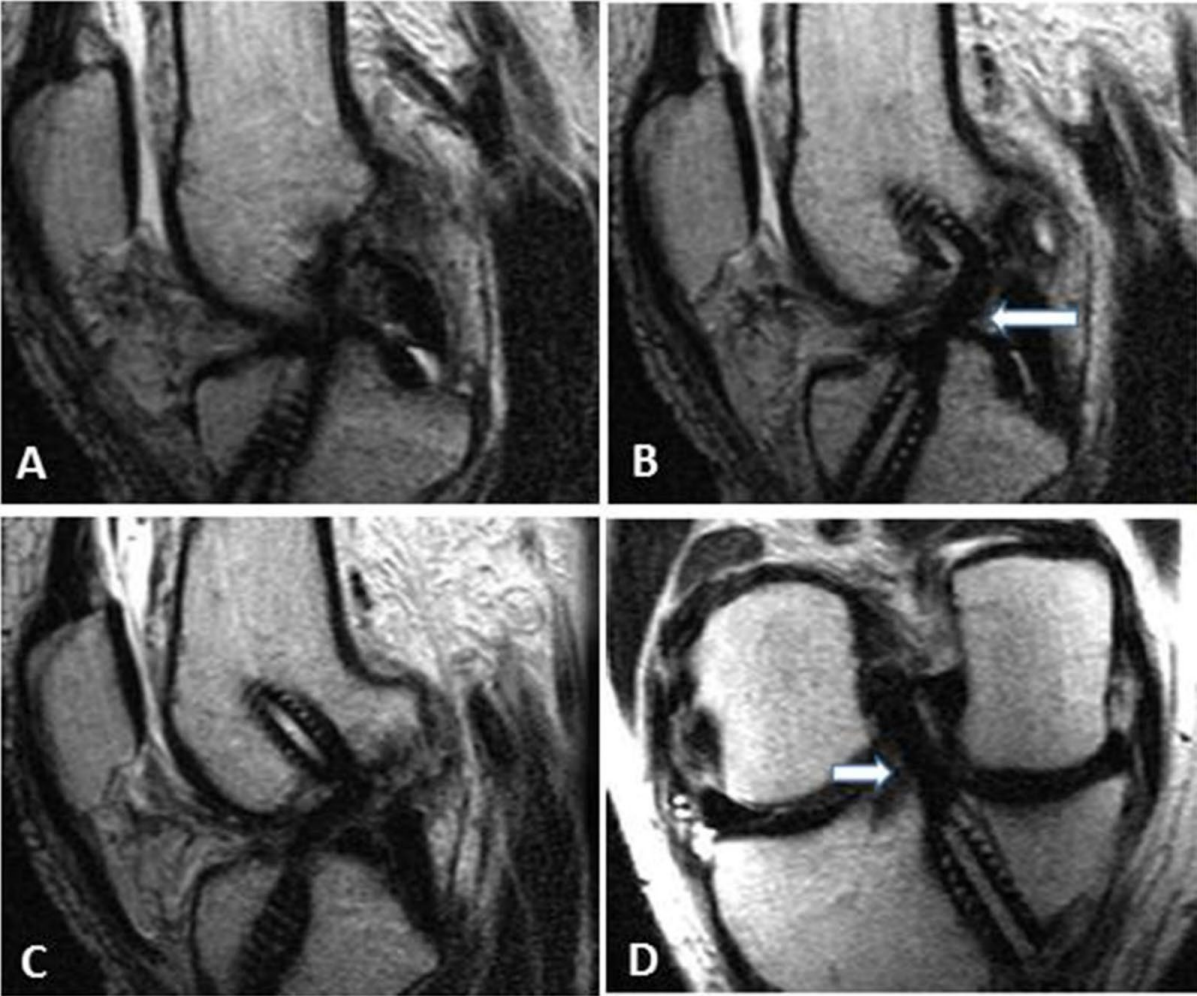
**a**, **b**, **c** Sagittal oblique T2 WIs and** d**, coronal oblique T2 WIs (protocol B) of the same patient mentioned in Fig. 7, showing intact (grade 0) graft as a low signal intensity graft with well-preserved continuation and a taut orientation (thick white graft). Arthroscopy confirmed the intact nature of the graft

Regarding protocol B, Reader 1 and arthroscopy had matching results in 25 cases and disagreed in 9 cases. Among the 25 cases with matching results, 7 cases had complete tear, 3 cases had partial tear and 15 cases had no tear. Reader 2 and arthroscopy had matching results in 26 cases and disagreed in 10 cases. Among the 26 cases with matching results: 7 patients had complete tear, 2 patients had partial tear and 17 patients had no tear **(**Figs. 7, 8).

The interobserver agreement amid the two readers was ranked as “**moderate**” for imaging with protocol A with weighted kappa value of 0.606 and “**substantial**” for imaging with protocol B with weighted kappa value of 0.759. These findings are summarized in Tables 7 and 8.

**Table 7:** Interobserver agreement between the two readers in imaging with protocol A

Protocol A	Reader 1					
	Complete tear	Partial tear	No tear	Total	Kappa	95%CI
*Reader 2*						
Complete tear	7	0	0	7	0.606	0.395–0.818
Partial tear	3	5	0	8		
No tear	0	6	15	21		
Total	10	11	15	36

**Table 8:** Interobserver agreement between the two readers in imaging with protocol B

Protocol B	Reader 1					
	Complete tear	Partial tear	No tear	Total	Kappa	95%CI
*Reader 2*						
Complete tear	8	0	0	8	0.759	0.570–0.948
Partial tear	1	4	0	5		
No tear	0	4	19	23		
Total	9	8	19	36		

**Fig. 9:**
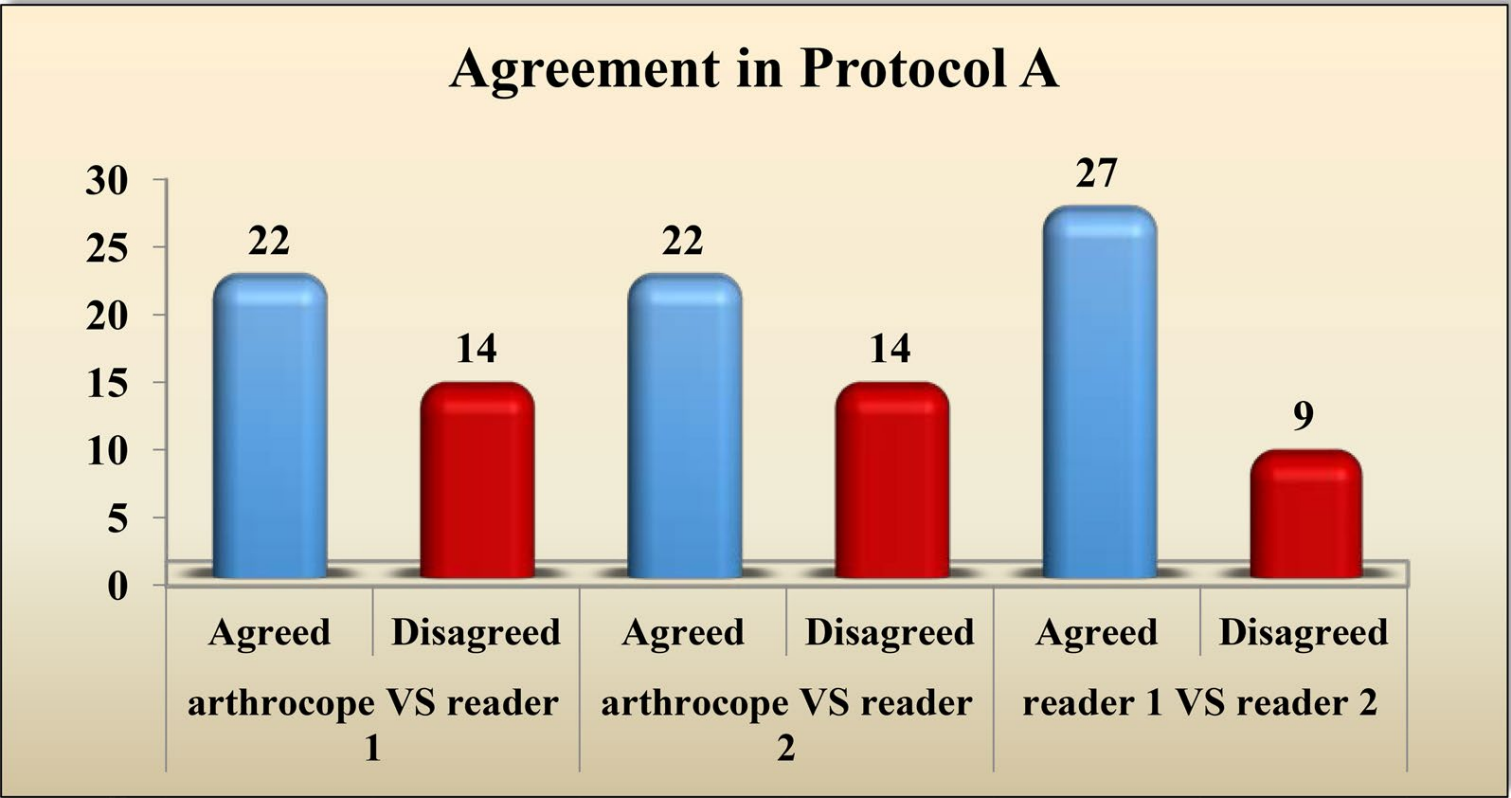
The agreement between each reader and the arthroscopy, in addition to the interobserver agreement for protocol A

**Fig. 10:**
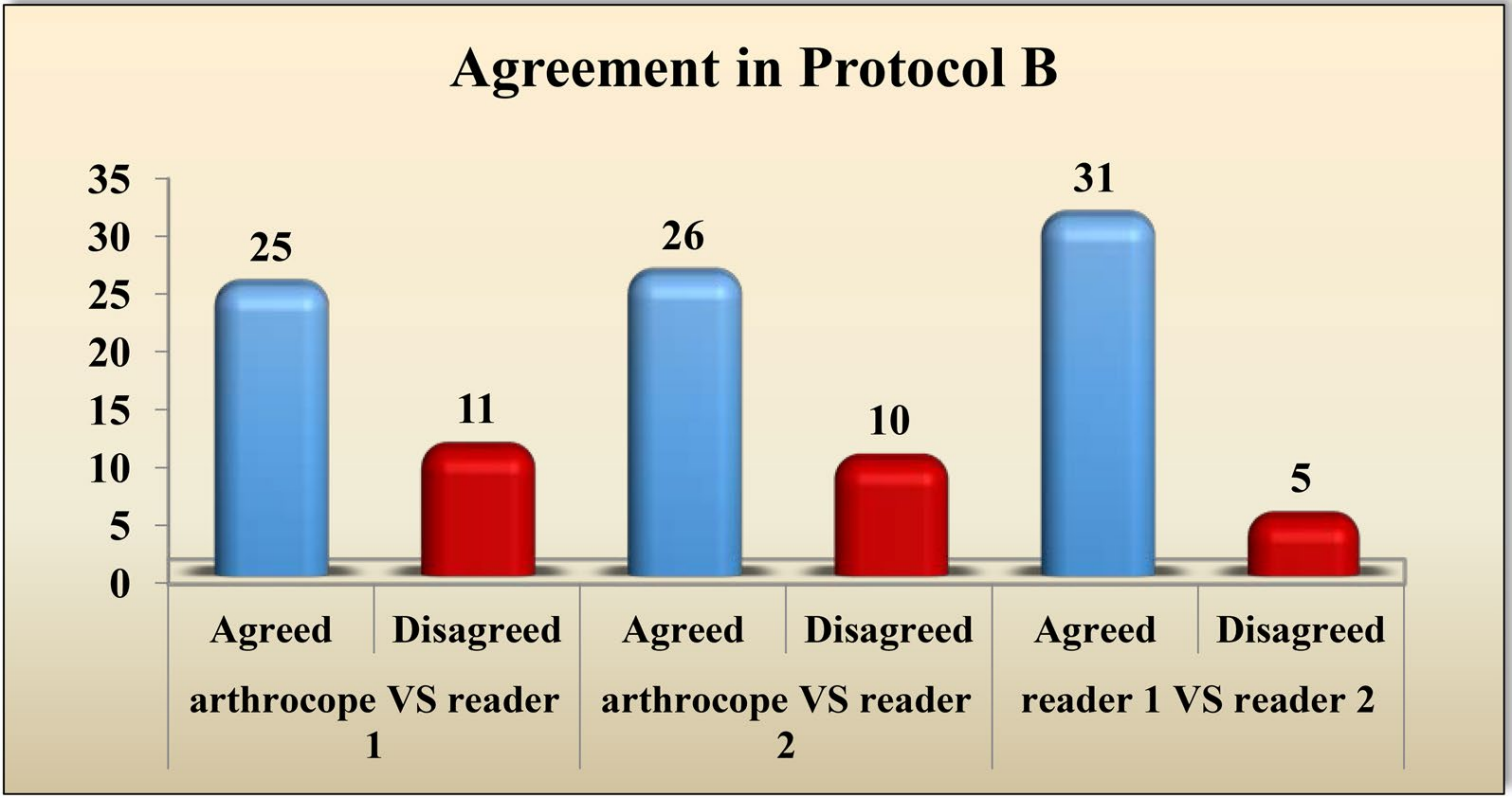
The agreement between each reader and the arthroscopy, in addition to the interobserver agreement for protocol B

The agreement between each reader and the arthroscopy, in addition to the interobserver agreement for protocols A and B, is illustrated in Figs. 9 and 10, respectively.

The MR sensitivity, specificity and accuracy for the diagnosis of ACL graft partial tear and complete tear were evaluated as shown in Table 9. The imaging with protocol B had higher specificity and accuracy than did the imaging with protocol A for each reader.

**Table 9:** The MR sensitivity, specificity and accuracy for the diagnosis of ACL graft partial and complete tear

	Sensitivity (%)	Specificity (%)	Accuracy (%)
*Reader 1 in protocol A*			
Complete tear	70.0	66.7	67.9
Partial tear	37.5	66.7	57.7
*Reader 2 in protocol A*			
Complete tear	60.0	77.8	71.4
Partial tear	25.0	77.8	61.5
*Reader 1 in protocol B*			
Complete tear	70.0	83.3	78.6
Partial tear	37.5	83.3	69.2
*Reader 2 in protocol B*			
Complete tear	70.0	94.4	85.7
Partial tear	25.0	94.4	73.1

For diagnosing partial ACL graft tear by reader 1, the addition of oblique views increased the specificity and accuracy from 66.7 and 57.7 to 83.3% and 69.2%, respectively. For diagnosing partial ACL graft tear by reader 2, the addition oblique views increased the specificity and accuracy of the examination from 77.8 and 61.5 to 94.4% and 73.1%, respectively. For diagnosing of a complete ACL graft tear by reader 1, the addition of oblique views increased the specificity and accuracy from 66.7 and 66.9 to 83.3% and 78.6%, respectively. For the diagnosis of a complete ACL graft tear by reader 2, the addition oblique views increased the specificity and accuracy of the examination from 77.8 and 71.4 to 94.4% and 85.7%, respectively. However, the sensitivity remained low for diagnosing ACL graft partial and complete tear using both MRI protocols A and B with both readers.

## Discussion

In our study, the MR specificity and diagnostic accuracy for evaluation of ACL graft tears were enhanced by adding the oblique coronal and oblique sagittal images.

Moon et al. [8] found similar results and they stated that the diagnostic accuracy for ACL graft injury was enhanced by the adding oblique coronal images to the standard knee MR sequences. Several studies have used oblique images of the knee MRI in the evaluation of healthy ACL grafts after double-bundle or selective-bundle ACL reconstructions [14, 15]. Casagranda et al. [14] used only the oblique coronal view, while Kiekara et al. [15] used both oblique sagittal and coronal views for evaluation of ACL graft.

We made benefit of adding the oblique coronal images as Moon et al. [8] who attributed the superior abilities of full length discrimination of the oblique view to the fact that, the oblique coronal angle is similar to the oblique lie of the ACL graft, which is less subject to volume averaging. Moon et al. also proposed that oblique coronal views improve the visualization of the transverse width of the ACL graft because both the medial and lateral margins of the graft are properly seen. Finally, he stated that oblique images decrease paramagnetic artifacts by avoiding fixation devices in the plane, while artifacts from these devices obscure the femoral and tibial bone tunnel images on the orthogonal view.

To overcome a false diagnoses for ACL graft injury made with using the oblique coronal images, we used also the oblique sagittal images to enhance proper visualization of the femoral attachment site of an ACL graft by showing the femoral tunnel in a plane. In our study, we thought that the femoral attachment site is vulnerable to misinterpretation because of the acute angle formed between the femoral tunnel and the grafts on the oblique coronal images. This addition was inspired by the study done by Horton et al. [16].

In our study, we used a 1.5-Tesla MRI scanner which we think it could provide more information about an ACL injury than a less available and probably more expensive study performed on a 3-Tesla scanner. Other previous studies evaluated also the role of 1.5-Tesla MRI in evaluation of ACL and ACL graft [6, 17]. On the other hand, many other recent studies have discussed the role of 3-Tesla MRI in evaluation of ACL grafts [18, 19].

Not only we evaluated the role of oblique sagittal and oblique coronal MRI for assessment of ACL graft injury, but we also evaluated their role in grading the severity of ACL graft injury. Teraoka et al. [18] and Song et al. [17] used a grading system inspired by the one used by Hong et al. [20], as they classified the subjects into three grades based on the MRI findings: grade 1, with low-intensity signal of the graft; grade 2, with high-intensity signal within 50% of the graft; and grade 3, with high-intensity signal greater than 50% of the graft.

In our study, we found that, while the addition of oblique coronal and sagittal MRI to conventional MRI images increased the specificity and accuracy for detection of ACL graft partial and complete tear, the sensitivity remains low. Kim et al. [7] combined partial tear and complete tear of ACL graft under the term of “ACL graft failure.” They stated that addition of the ACL views to the orthogonal view might be more specific and accurate than the orthogonal views only for the diagnosis of double-bundle ACL graft failure. They also stated that the sensitivities by using additional views rather decreased slightly. They attributed this finding to the fact that too many images can mask subtle signal change of the ligament. The diagnosis of partial tear of an ACL graft is more challenging than that of complete tear. In a previously done MR study of 16 patients, the diagnosis of partial tear versus other conditions (intact graft or complete tear) resulted in 0% sensitivity, 67% specificity and 37.5% accuracy [16]. The readers in this previously done study made many false-negative diagnoses even when they used the oblique coronal images, because the MR interpretation was based on the morphologic abnormalities, and there was not enough information on functional abnormalities such as graft laxity. For these cases, we may increase the sensitivity of MR study by combining the information given by MR images with the provided clinical data.

In our study, the images were evaluated by two musculoskeletal radiologists blinded to the results of each other. A similar approach was done by many other studies [7, 8, 17].

In our study, we evaluated two imaging groups, protocol A, which included the standard MRI knee protocol and protocol B, which included the standard knee protocol together with the oblique images. Other studies followed the same approach for evaluation of oblique MRI images in evaluation of ACL graft failure [5, 8].

## Limitations

Our study had several limitations including the small sample size and the delay of the revision arthroscopy after the MR examination was done, as we encountered the spread of COVID-19 disease during cases collection with the elective surgeries (arthroscopy) being postponed. Finally, our study did not include objective quantitative assessment of the anatomic identification in reconstructed ACL. Instead, we used a subjective scoring system.

## Conclusions

In conclusion, we found that adding oblique sagittal and oblique coronal images to the routine MRI of the knee added more diagnostic value in detecting and grading ACL graft injury. It increased specificity and accuracy of the MRI examination compared with using the routine MRI of the knee alone, with the arthroscopy used as a reference gold standard. Such addition to the MRI knee protocol may help the surgeons to choose the proper management in cases with suspected ACL graft failure.

## Data Availability

The datasets used and analyzed during the current study are available from the corresponding author on reasonable request.

## Abbreviations

ACL: Anterior cruciate ligament
MRI: Magnetic resonance imaging
CI: Confidence interval
